# Mechanism of Paeoniflorin on ANIT-Induced Cholestatic Liver Injury Using Integrated Metabolomics and Network Pharmacology

**DOI:** 10.3389/fphar.2021.737630

**Published:** 2021-08-30

**Authors:** Lisheng Chen, Xu Zhao, Shizhang Wei, Xiao Ma, Honghong Liu, Jianyu Li, Manyi Jing, Min Wang, Yanling Zhao

**Affiliations:** ^1^ Department of Pharmacy, Hebei North University, Zhangjiakou, China; ^2^ Department of Pharmacy, The Fifth Medical Center of Chinese PLA General Hospital, Beijing, China; ^3^ Hepotology Department, The Fifth Medical Center of Chinese PLA General Hospital, Beijing, China; ^4^ College of Pharmacy, Chengdu University of Traditional Chinese Medicine, Chengdu, China; ^5^ Integrated TCM and Western Medicine Department, The Fifth Medical Center of Chinese PLA General Hospital, Beijing, China

**Keywords:** paeoniflorin, cholestatic liver injury, feces, metabolomics, network pharmacology

## Abstract

**Background:** Paeoniflorin (PF), the major active compound isolated from the roots of *Paeonia lactiflora* Pall., has been used in the treatment of severe hepatic diseases for several decades and displays bright prospects in liver protective effect. However, its biological mechanism that regulates bile acid metabolism and cholestatic liver injury has not been fully elucidated. Our study aims to investigate the mechanism by which PF in the treatment of cholestatic liver injury using a comprehensive approach combining metabolomics and network pharmacological analysis.

**Methods:** The hepatoprotective effect of PF against cholestasis liver injury, induced by α-naphthylisothiocyanate (ANIT), was evaluated in rats. The serum biochemical indices including ALT, AST, TBA, TBIL, ALP, ALB, and the pathological characteristics of the liver were analyzed. Moreover, UHPLC-Q-TOF was performed to explore the feces of rats with ANIT-induced cholestatic liver injury treated with PF and the potential biomarkers were screened by metabolomics. The targets for the regulation of potential biomarkers by PF were screened by network pharmacology, and then the relevant key targets were verified by immunohistochemical and western blotting methods.

**Results:** PF significantly improved serum indexes and alleviated liver histological damage. Metabolomics analyses showed that the therapeutic effect of PF is mainly associated with the regulation of 13 metabolites involved in 16 metabolic pathways. The “PF-targets-metabolites” interaction network was constructed, and then five key targets including CDC25B, CYP2C9, MAOB, mTOR, and ABCB1 that regulated the potential biomarkers were obtained. The above five targets were further verified by immunohistochemistry and western blotting, and the results showed that PF significantly improved the expression of key proteins regulating these biomarkers.

**Conclusion:** Our study provides direct evidence for the modulatory properties of PF treatment on ANIT-induced cholestatic liver injury using metabolomics and network pharmacology analyses. PF exhibits favorable pharmacological effect by regulating related signal pathways and key targets for biomarkers. Therefore, these findings may help better understand the complex mechanisms and provide a new and effective approach to the treatment of cholestatic liver injury.

## Introduction

Cholestasis is a pathological process caused by the disturbance of bile secretion and excretion, which is characterized by excessive accumulation of bile acids, cholesterol, bilirubin and other bile components in the liver and systemic circulation, leading to liver lesions (Chen et al., 2018). Cholestasis occurs in people of any age and sex, and a variety of factors can cause cholestasis, such as drug-induced liver injury (DILI), heredity (progressive familial intrahepatic cholestasis) and diseases (septicemia, cholelithiasis). If left untreated, cholestasis will further develop into liver fibrosis, cirrhosis and even liver cancer (Jansen et al., 2017). Due to the complex etiology and injury mechanism of cholestasis, the development of new drugs related to cholestasis is seriously restricted. At present, the treatment of cholestasis is very limited. Ursodeoxycholic acid (UDCA) can treat primary biliary cholangitis (PBC), but more than 40% of patients respond poorly, about 10% of patients cannot tolerate it, and its effectiveness is limited to the early stage of PBC. Obecholic acid (OCA) can significantly improve the biochemical indexes, delay the disease progression and improve the survival rate of PBC patients who do not respond well or tolerate UDCA treatment, but it produces serious side effects such as pruritus during OCA treatment, and excessive use of OCA aggravates liver injury (Kowdley et al., 2018). Therefore, the search for drugs related to the treatment of cholestatic liver injury has been the focus of research in recent years.

Paeoniflorin, a monoterpenoid glycoside compound, is the main active ingredient of the *Paeonia lactiflora* Pall.. In recent years, a large number of studies have shown that PF has the effects of anti-inflammation (Tu et al., 2019), anti-oxidation (Zhao et al., 2013), anti-depression (Cheng et al., 2021), anti-tumor (Zhang et al., 2018), immune regulation (Kong et al., 2018) and liver protection (Wei et al., 2020), and has less toxic and side effects, so its medicinal value has been paid more and more attention. However, the potential mechanism of PF in relieving cholestatic liver injury is not clear, and whether PF can be safely and effectively used in clinic still needs further study. A variety of constructive technologies such as, proteomics, genome chip and network pharmacology have been used to explore the action mechanism and active substances of traditional Chinese medicine in the past few decades (Xu et al., 2017). However, few researchers have fully integrated a variety of techniques to explore the mechanism of PF in the treatment of cholestatic liver injury.

Traditional Chinese medicine has the synergistic regulation of multi-components, multi-targets and multi-pathways, so it is difficult to clarify the mechanism of liver protection, which hinders the development of clinical treatment of liver disease with traditional Chinese medicine. Metabolomics is an effective method to study the mechanism of liver protection of traditional Chinese medicine. It is a new subject after proteomics and genomics in recent years, which is also one of the hot research fields in recent years. Metabolomics comprehensively reflects the changes of endogenous differential metabolites before and after liver injury, deeply analyzes the metabolic pathways related to biomarkers, and elucidates the mechanism of liver protection. In addition, the potential therapeutic targets of various types of liver injury can be obtained through the analysis of signal pathways, proteins and genes closely related to metabolic pathways in collaboration with other disciplines, which provides many useful clues for the in-depth understanding of various physiological and pathological phenomena of the body and the pathogenesis of diseases (Wang et al., 2011; Xi-lan, 2021; Xiong et al., 2014). In addition, network pharmacology, as an effective tool to predict and reveal the complex relationship between targets, diseases and drugs, has been successfully applied to understand the components of traditional Chinese medicine and the mechanism of diseases. Therefore, the combination of metabolomics and network pharmacology further reveals the molecular mechanism of disease regulation (Lyu et al., 2018; Zhang et al., 2019).

Recently, it has been reported that endogenous bile acid hepatointestinal circulation is involved in the pathogenesis of cholestatic liver disease (Blesl and Stadlbauer, 2021). Intestinal bacteria convert primary bile acid excreted from the liver into secondary bile acid in the intestine, and then 95% of the bile acid can be reabsorbed by the intestinal wall. The liver can regulate intestinal metabolism by secreting metabolites such as bile acids. Therefore, the composition of intestinal metabolites may play a key role in intestinal-liver axis crosstalk (Marrero et al., 2018; Milosevic et al., 2019). In this study, the potential biomarkers were obtained by metabonomic analysis of rat feces, and the targets of biomarkers were screened and verified by network pharmacology and molecular biology methods. Therefore, we elucidated the potential fecal biomarkers and possible mechanisms of action of PF in the treatment of cholestatic liver injury, which might provide novel insight into improving the treatment of cholestasis (Supplementary Figure S1).

## Materials and Methods

### Chemicals and Reagents

Paeoniflorin (C_23_H_28_O_11_, purity >98%, Cat. No. J0T-10063) was purchased from Chengdu Pufei De Biotech Co., Ltd. (Chengdu, China). ANIT was purchased from Sigma Chemical Co. (St. Louis, MO, United States). Ursodeoxycholic acid (UDCA) was obtained from Losan Pharma GmbH (Germany). The alanine aminotransferase (ALT), aspartate transaminase (AST), total bile acid (TBA), total bilirubin (TBIL), alkaline phosphatase (ALP), and albumin (ALB) kits were purchased from Nanjing Jiancheng Bioengineering Institute (Nanjing, China). All of the other experimental supplies were commercially available.

### Animals

Male Sprague-Dawley rats weighing 190 ± 10 g were obtained from Sibeifu (Beijing) Biotechnology Co., Ltd. [Beijing, China, Permission No. SCXK (jing) 2019-0010]. All animals were kept at the specific environment (humidity: 55 ± 5%, temperature: 25°C ± 0.5°C and 12 h/12 h light/dark cycle) and given sufficient sterile food and water. All animal experiments were in accordance with the Animal Experimental Ethics Committee of the Fifth Medical Center of PLA General Hospital (approved ID: IACUC-2019-004).

All rats were allowed to acclimate for 1 week prior to the experiment and were randomly divided into five groups of six rats each: (A) Control group; (B) ANIT group; (C) UDCA group (60 mg/kg); (D) Paeoniflorin low dose group (PFL, 50 mg/kg); (E) Paeoniflorin high dose group (PFH, 200 mg/kg). The PF was dissolved in normal saline and intragastrically administered to rats for 5 days. The rats in the control group were received with normal saline every day and were intragastric administrated with vehicle (olive oil) alone. Rats in the ANIT group, PFL group, PFH group, and UDCA group intragastrically received 60 mg/kg ANIT (dissolved in olive oil) on the third day. UDCA, the positive drug, was given to rats for 5 days using the same conditions used for the PF group.

### Sample Collection and Preparation

Before the end of the experiment, each rat was put into a metabolic cage (1 per cage) to collect feces for 12 h, and then the rats were anaesthetized with 20% ethyl carbamate solution and blood was taken from the abdominal aorta. Then, the liver samples were rapidly excised and fixed in 10% paraformaldehyde solution for histopathological analysis. The blood was centrifuged at 3,000 rpm for 10 min to separate the serum and transferred to −80°C for preservation, and then the serum liver function indexes were determined.

### Analysis of Serum Biochemical Indexes

The serum levels of AST, ALT, TBA, TBIL, ALP, and ALB were measured by commercial test kits (Nanjing Jiancheng, Nanjing, China) according to the manufacturer’s instructions.

### Histological Examination

The liver specimen was fixed in 10% formalin solution, embedded in paraffin wax and sliced into 5 μm sections. Deparaffinized sections were stained with hematoxylin-eosin (HE staining) and examined by light microscopy as described previously (Zhu and Feng, 2019).

### Sample Handling

Briefly, the fecal samples from the control group, ANIT group, and PFH group were thawed at room temperature. Then 50 mg faeces from each rat was added to 1 ml methanol solution and then underwent an extraction with vigorous shaking for 2 min. The samples were centrifuged at 12,000 rpm at 4°C for 10 min to remove any solid debris. The supernatant was transferred to a new 1.5 ml centrifuge tube and then filtered through a syringe filter (0.22 µm) to obtain the sample for injection as described previously (Tian et al., 2015).

### Chromatography and Mass Spectrometry

The Agilent 1,290 series UPLC system equipped with quaternary pump, online degasser, autosampler, and thermostat column compartment was used for fecal metabolic profiling analysis. The sample injection volume was 4 µL and the flow rate was 0.30 ml/min, and all the samples were performed on a ZORBAX RRHD 300 SB-C18 column (2.1 mm × 100 mm, 1.8 μm, Agilent Technologies, Santa Clara, CA, United States) at 4°C. The column temperature was set at 30°C. The mobile phases were composed of 0.1% formic acid in acetonitrile (solvent A) and 0.1% formic acid in water (solvent B). The gradient program was consistent with the previous report (Chen et al., 2020). QC sample compounded with all samples was injected to ensure the stability and repeatability of the systems after injection of the 10 samples.

Mass spectrometry was carried out on an Agilent 6550A Q-TOF/MS (Agilent Technologies, Santa Clara, CA, United States) with an electrospray ionization source (ESI) in both positive and negative mode. The electrospray source parameters were fixed as follows: electrospray capillary voltage: 3.0 kV in the negative ionization mode and 4.0 kV in the positive ionization mode; gas temperature: 200°C in the negative ionization mode and 225°C in the positive ionization mode; nozzle voltage: 500 V in both ionization modes; gas flow: 11 L/min; nebulizer: 35 pisg (negative) and 45 pisg (positive); mass range: 80–1,000 m/z.

### Data Extraction and Pattern Recognition Analysis

The sample data were extracted by using MassHunter Profinder software (Agilent, California, United States). The initial and final retention times were set for data collection. Data were normalized with MetaboAnalyst 5.0 and then the resultant data matrices were introduced to SIMCA-P 14.1 software (Umetrics, Umea, Sweden) for principal component analysis (PCA) and orthogonal-partial least squares discriminant analysis (OPLS-DA). These variables with VIP >1.5 and |p (corr)| ≥ 0.58 in the OPLS-DA method were considered to be further data analysis.

### Potential Metabolites Identification and Pathway Enrichment Analysis

Components that changed significantly between groups were identified as biomarkers. The potential biomarkers were identified by the online biochemical database service METLIN (http://metlin.scripps.edu/) and HMDB database (http://www.hmdb.ca/). MetaboAnalyst 5.0 (https://www.metaboanalyst.ca/) was used for further enrichment and pathway analysis of the previously identified potential metabolites.

### Identification of PF Targets and Potential Metabolites

To identify the corresponding relationships between potential biomarkers and their related targets, we performed network analysis using network pharmacology. PharmMapper Server (Version 2017) was used to screen PF drug targets and the MBrole 2.0 database was used to screen the corresponding targets of identified potential biomarkers. Interacting protein targets were screened using the Interacting Protein Database (DIP). Then, all the protein ID types of the obtained targets were converted to UniProt IDs and the “PF-target-potential biomarker” interactive network was established by using Cytoscape 3.7.2 (National Institute of General Medical Sciences, United States).

### Western Blotting

Rat liver tissue (80 mg) were homogenized and then lysed in the prepared ice-cold lysis buffer with 1 mM phenylmethylsulfonyl fluoride and a protease inhibitor mixture, and then centrifuged at 12,000 × g and 4°C for 10 min. The supernatant was collected and the BCA Protein Assay Kit (Beijing Solarbio Science & Technology Co., Ltd., Beijing, China) was used for quantification. All prepared samples were western blotted with 10% sodium dodecyl sulfatepolyacrylamide gel electrophoresis (SDS-PAGE) and then transferred to polyvinylidene fluoride (PVDF) membrane. The PVDF membranes were blocked with 5% fat-free milk at room temperature for 2 hours, then incubated overnight at 4°C with antibodies against anit-CDC25B (PB9488, BOSTER, dilution: 1:1,000), anit-MTOR (A00003-2, BOSTER, dilution: 1:1,000), anit-CYP2C9 (16546-1-AP, proteintech, dilution: 1:2,000), anit-ABCB1 (A00049-3, BOSTER, dilution: 1:1,000), anit-MAOB (PB9665, BOSTER, dilution: 1:1,000). Then wash with TBS-0.1% Tween 20 (TBST) 2 times for 5 min each time, and incubate with horseradish peroxidase conjugated secondary antibodies at room temperature for 1 h. The immunoreactive bands were visualized with chemiluminescence.

### Immunohistochemistry

In order to evaluate the effects of PF on key proteins regulating fecal metabolism in rats with cholestasis liver injury, the liver tissue of rats was detected by immunohistochemical method as described previously (Wei et al., 2020). The liver tissue slides were incubated with anit-CDC25B, anit-MTOR, anit-CYP2C9, anit-ABCB1, anit-MAOB for 1.5 h, followed by incubation with peroxidase-coupled secondary antibody for 30 min. Sections were incubated with streptavidin-peroxidase-biotin complex for 20 min at room temperature and then incubated with 3,3′-diaminobenzidine hydrochloride for 5 min for color development. The positive areas showed the color of brown yellow. The images were examined using a digital camera system under 200× magnification and analyzed by using Image J 1.48v.

### Statistical Analysis

Statistical analysis was performed by using SPSS 20.0 software program (Chicago, United States) and GraphPad Prism 8.02 software (San Diego, United States) was used to visualize the results. All data were presented as the mean ±SD. The differences between the group means were calculated by ANOVA. *p* < 0.05 was considered statistically significant, and *p* < 0.01 was considered highly significant.

## Results

### Effects of PF on ANIT-Induced Cholestatic Liver Injury in Rats

**FIGURE 1:**
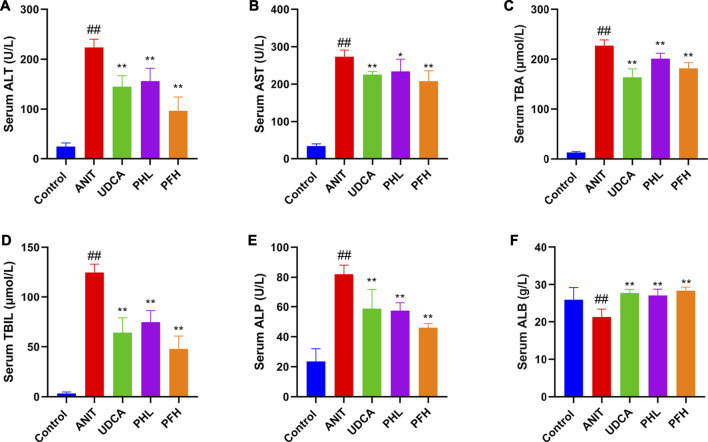
Effect of paeoniflorin on serum biochemical indexes. **(A)** serum ALT level; **(B)** serum AST level; **(C)** serum TBA level; **(D)** serum TBIL level; **(E)** serum ALP level; **(F)** serum ALB level. Data were presented as means ± SD (*n* = 6). *##* *p <* 0.01, *#* *p <* 0.05 compared with the control group, ** *p <* 0.01, * *p <* 0.05 compared with ANIT group.

To evaluate the protective effect of PF on cholestatic hepatocyte injury, the serum biochemical indexes related to liver injury and cholestasis were detected. As shown in Figures 1A–F, the serum levels of ALT, AST, TBA, TBIL, and ALP exhibited the remarkable increases in ANIT-induced cholestasis rats. Conversely, the levels of ALT, AST, TBA, TBIL, and ALP were reduced after treatment with the PF, which coincided with the findings of our previous study (Chen et al., 2016). Furthermore, ANIT administration significantly reduced the serum level of ALB. However, PF (200 mg/kg and 50 mg/kg) improved the reduction of ALB, which was similar to UDCA.

**FIGURE 2:**
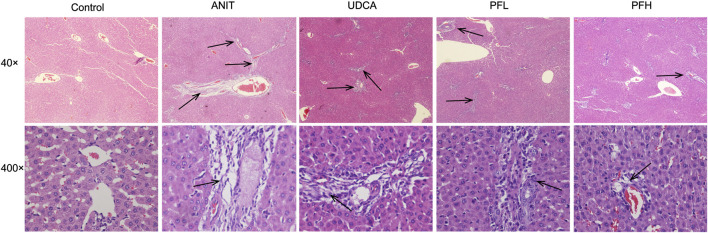
Effect of PF on the histopathology of liver tissues using HE staining (40× and 400×). The groups were control group, ANIT group, UDCA group, PFL group and PFH group. Inflammatory infiltration, destructive interlobular ducts, and hepatic necrosis as shown by the arrow.

Histological evaluations provided visual evidence for the protective effect of PF on ANIT-induced cholestatic liver injury. Liver histopathology showed that the liver tissue of control group exhibited a normal structure with large and round hepatic cell nucleus and evident nuclear membrane, while ANIT-treated rat liver specimens showed inflammatory infiltration, destructive interlobular ducts, and hepatic necrosis. This result is consistent with our previous study (Zhao et al., 2017). However, rats treated with UDCA and PFL exhibited weak attenuation of inflammatory infiltration and bile duct epithelial damage. On the contrary, the specimens from PFH group displayed potent decrease in inflammatory cell infiltration, destructive interlobular ducts and necrosis. These results indicated that PF significantly protected against ANIT-induced cholestatic liver injury (Figure 2).

### Multivariate Statistical Analysis

**FIGURE 3:**
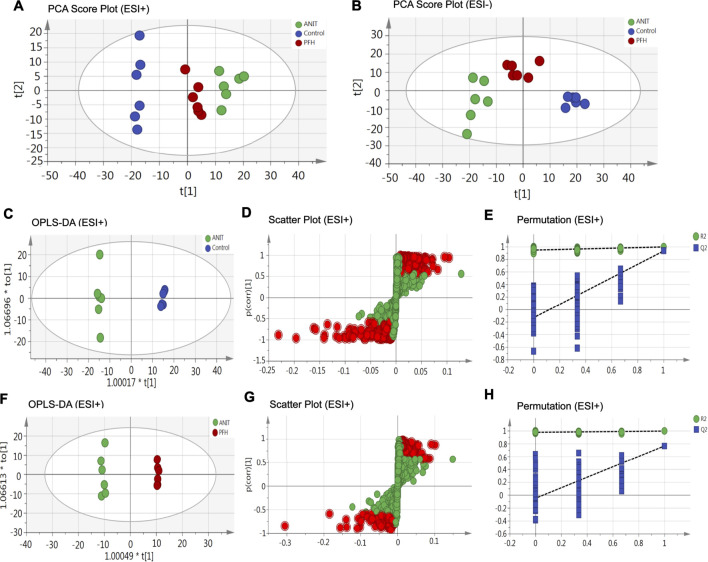
The principal component analysis (PCA) score plot of fecal metabolomics analysis in the ESI+ model **(A)** and ESI− model **(B)**. OPLS-DA score plots were the pair-wise comparisons between the control and ANIT groups **(C)** as well as between the ANIT and PFH groups **(F)**. S-plots of the OPLS-DA model for the control and ANIT groups **(D)** as well as for the ANIT and PFH groups **(G)**. The 100-permutation test of the OPLS-DA model was for the control and ANIT groups **(E)** as well as for the ANIT and PFH groups **(H)**.

To reveal the mechanism of PF in the treatment of cholestasis, we used metabolomics to detect the effect of PF on fecal endogenous metabolism in rats with cholestasis. Principal component analysis (PCA) was originally used as an unsupervised statistical method to summarize and distinguish the metabolic phenotypes and metabolites among the control group, ANIT group, and 200 mg/kg PF group in both ESI+ and ESI− models. The score plot provided a direct image of observational clusters. As shown in Figures 3A,B, the clustering significantly distinguished between control group, ANIT group and PFH group in both the positive and negative modes, which indicated ANIT-induced remarkable changes in fecal endogenous metabolites and PF could restore the metabolic profiling of fecal in cholestasis rats.

OPLS-DA was used to better understand the different metabolic patterns and identify potential metabolites that were significantly changed between the control, ANIT and PFH groups. The R^2^X (cum), R^2^Y (cum), and Q^2^ (cum) values provide an estimate of how well the model fits the data. The R^2^X (cum), R^2^Y (cum), and Q^2^ (cum) of OPLS-DA in our positive model were 0.405, 0.999, and 0.932, respectively, using the data from the control and ANIT groups and 0.28, 0.998, and 0.767, respectively, using the data from the ANIT and PFH groups (Figures 3C,F). The R^2^X (cum), R^2^Y (cum), and Q^2^ (cum) of OPLS-DA in our negative model were 0.435, 0.999, and 0.946, respectively, using the data from the control and ANIT groups and 0.308, 0.995, and 0.79, respectively, using the data from the ANIT and PFH groups (Supplementary Figure S2). These data indicated that models were of good quality and provided accurate predictions. Variables with distant free points were thought to contribute more to the separation between different groups; therefore, they were identified as potential metabolites in both the positive (Figures 3D,G) and negative models (Supplementary Figure S2). To verify the validity of the data multivariate analysis model, permutation tests with 200 iterations were further carried out. The validation plots indicated that the original models were valid in the positive (Figures 3E,H) and negative modes (Supplementary Figure S2).

### Identification of Potential Metabolite of PF in the Treatment of ANIT-Induced Cholestatic Liver Injury

**FIGURE 4:**
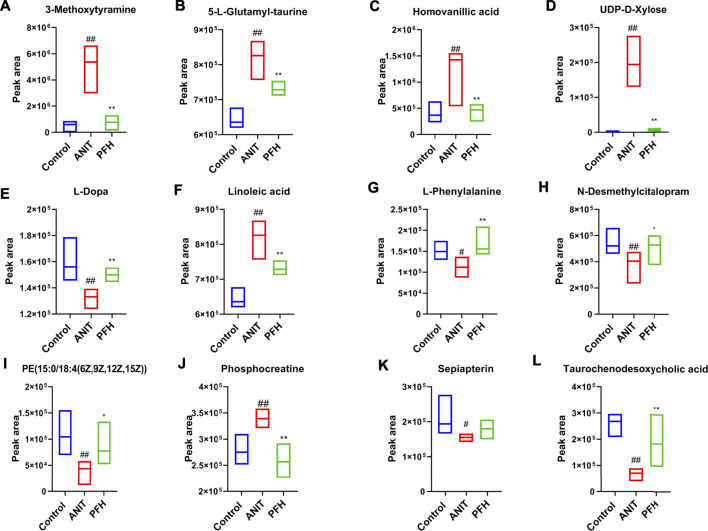
Potential metabolites changes in ANIT-induced cholestatic liver injury with PF treatment. **(A)** 3-Methoxytyramine; **(B)** 5-L-Glutamyl-taurine; **(C)** Homovanillic acid; **(D)** UDP-D-Xylose; **(E)** L-Dopa; **(F)** Linoleic acid; **(G)** L-Phenylalanine; **(H)** N-Desmethylcitalopram; **(I)** PE [15:0/18:4 (6Z,9Z,12Z,15Z)]; **(J)** Phosphocreatine; **(K)** Sepiapterin; **(L)** Taurochenodesoxycholic acid. Data were presented as means ± SD (*n* = 6). ^*##*^ *p <* 0.01, ^*#*^ *p <* 0.05 compared with the control group, ^**^ *p <* 0.01, ^*^ *p <* 0.05 compared with ANIT group.

Metabolites that meet a threshold of VIP ≥1.5 and |P (corr)| ≥ 0.58 were considered potential candidates according to OPLS-DA analysis. The candidates that significantly changed among the groups with a *p*-value below 0.05 were identified as potential metabolites for METLIN and Metaboanalyst databases. It was found that 13 specific metabolites were related to the treatment of cholestasis with PF and relevant information and variation trends between groups were listed in Table 1. The significantly changed components were shown in Figure 4.

**TABLE 1:** The biomarkers of PF in the treatment of cholestasis.

No	KEGG ID	Compound name	Formula	Monoisotopic_mass	Pathway name
1	C01272	Inositol 1,3,4,5-Tetrakisphosphate	C_6_H_16_O_18_P_4_	499.9287098	Phosphatidylinositol signaling system; Inositol phosphate metabolism
2	C00835	Sepiapterin	C_9_H_11_N_5_O_3_	237.0861892	Folate biosynthesis
3	C16608	N-Desmethylcitalopram	C_19_H_19_FN_2_O	310.1481414	Drug metabolism-cytochrome P450
4	C00190	UDP-D-Xylose	C_16_H_20_N_2_O_7_	352.127051	Amino sugar and nucleotide sugar metabolism
5	C05465	Taurochenodesoxycholic acid	C_21_H_34_O_5_	366.2406242	Primary bile acid biosynthesis
6	C00355	L-Dopa	C_9_H_11_NO_4_	197.0688078	Tyrosine metabolism
7	C05587	3-Methoxytyramine	C_9_H_13_NO_2_	167.0946287	Tyrosine metabolism
8	C05582	Homovanillic acid	C_9_H_10_O_4_	182.0579088	Tyrosine metabolism
9	C00079	L-Phenylalanine	C_9_H_11_NO_2_	165.0789786	Phenylalanine, tyrosine and tryptophan biosynthesis; Phenylalanine metabolism; Aminoacyl-tRNA biosynthesis
10	C01595	Linoleic acid	C_18_H_32_O_2_	280.2402303	Linoleic acid metabolism; Biosynthesis of unsaturated fatty acids
11	C05844	5-L-Glutamyl-taurine	C_7_H_14_N_2_O_6_S	254.0572569	Taurine and hypotaurine metabolism
12	C00350	PE [15:0/18:4 (6Z,9Z,12Z,15Z)]	C_38_H_68_NO_8_P	697.4682547	Glycosylphosphatidylinositol (GPI)-anchor biosynthesis; Glycerophospholipid metabolism
13	C02305	Phosphocreatine	C_4_H_10_N_3_O_5_P	211.035807	Arginine and proline metabolism

### Pathway Analysis of PF in the Treatment of ANIT-Induced Cholestatic Liver Injury

**FIGURE 5:**
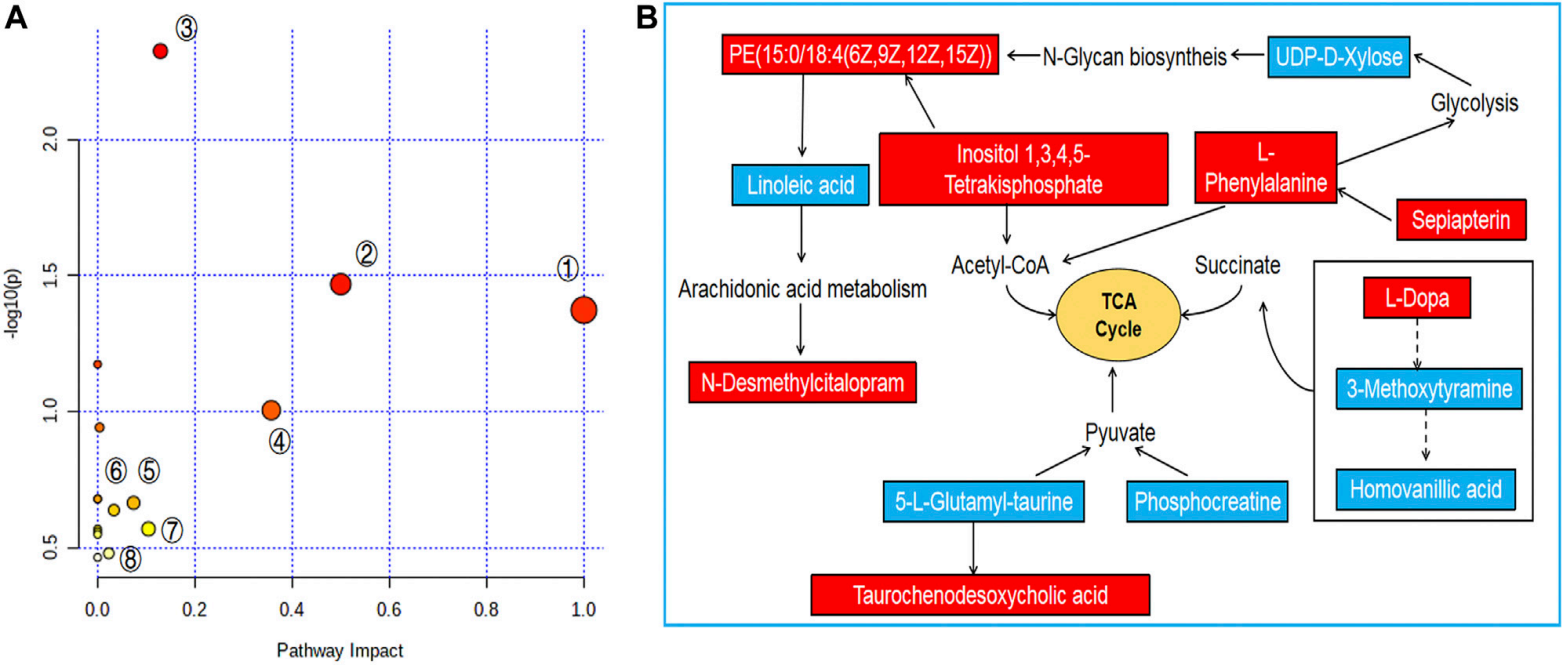
Pathway analysis of PF treatment. **(A)** Pathway impact by PF in the treatment of ANIT-induced cholestasis①: Linoleic acid metabolism; ②: Phenylalanine, tyrosine and tryptophan biosynthesis; ③: Tyrosine metabolism; ④: Phenylalanine metabolism; ⑤: Phosphatidylinositol signaling system; ⑥: Inositol phosphate metabolism; ⑦: Glycerophospholipid metabolism; ⑧: Primary bile acid biosynthesis. **(B)** Signaling networks associated with the differentially expressed metabolites pathways. The red solid box represents the peak area of the PFH/ANIT >1. The blue solid box represents the peak area of PFH/ANIT <1.

To further reveal the metabolic pathways of potential metabolites related to PF in the treatment of cholestasis, MetaboAnalyst 5.0 was used to analyze the pathway to visualize the affected metabolic pathways. 16 signaling pathways were enriched: tyrosine metabolism, phenylalanine, tyrosine and tryptophan biosynthesis, linoleic acid metabolism, taurine and hypotaurine metabolism, phenylalanine metabolism, glycosylphosphatidylinositol (GPI)-anchor biosynthesis, folate biosynthesis, drug metabolism-cytochrome P450, phosphatidylinositol signaling system, inositol phosphate metabolism, biosynthesis of unsaturated fatty acids, glycerophospholipid metabolism, amino sugar and nucleotide sugar metabolism, arginine, and proline metabolism, primary bile acid biosynthesis, aminoacyl-tRNA biosynthesis (Table 2 and Figure 5). The top eight pathways that impacted the bubble map were linoleic acid metabolism, phenylalanine, tyrosine and tryptophan biosynthesis, phenylalanine metabolism, tyrosine metabolism, phosphatidylinositol signaling system, inositol phosphate metabolism, primary bile acid biosynthesis, glycosylphosphatidylinositol (GPI)-anchor biosynthesis. These metabolic pathways were mainly involved in amino acid metabolism, bile acid metabolism and inflammation-related metabolism.

**TABLE 2:** Results of integrating enrichment analysis of biomarkers with MetaboAnalyst 5.0.

Pathway name	Match status	p	−log(p)	Holm p	FDR	Impact
Tyrosine metabolism	3/42	0.0047249	2.3256	0.39689	0.39689	0.12913
Phenylalanine, tyrosine and tryptophan biosynthesis	1/4	0.034051	1.4679	1.0	1.0	1.0
Linoleic acid metabolism	1/5	0.042394	1.3727	1.0	1.0	1.0
Taurine and hypotaurine metabolism	1/8	0.067028	1.1737	1.0	1.0	0.0
Phenylalanine metabolism	1/12	0.098964	1.0045	1.0	1.0	0.35714
Glycosylphosphatidylinositol (GPI)-anchor biosynthesis	1/14	0.11455	0.941	1.0	1.0	0.00399
Folate biosynthesis	1/27	0.20995	0.67789	1.0	1.0	0.0
Drug metabolism-cytochrome P450	1/27	0.20995	0.67789	1.0	1.0	0.0
Phosphatidylinositol signaling system	1/28	0.21688	0.66378	1.0	1.0	0.07388
Inositol phosphate metabolism	1/30	0.23057	0.63719	1.0	1.0	0.03345
Biosynthesis of unsaturated fatty acids	1/36	0.27033	0.5681	1.0	1.0	0.0
Glycerophospholipid metabolism	1/36	0.27033	0.5681	1.0	1.0	0.0
Amino sugar and nucleotide sugar metabolism	1/37	0.27677	0.55787	1.0	1.0	0.0
Arginine and proline metabolism	1/38	0.28316	0.54797	1.0	1.0	0.0
Primary bile acid biosynthesis	1/46	0.33242	0.47832	1.0	1.0	0.02285
Aminoacyl-tRNA biosynthesis	1/48	0.34423	0.46315	1.0	1.0	0.0

### “Potential Biomarkers-Target-Component” Interactive Network and Analysis

**FIGURE 6:**
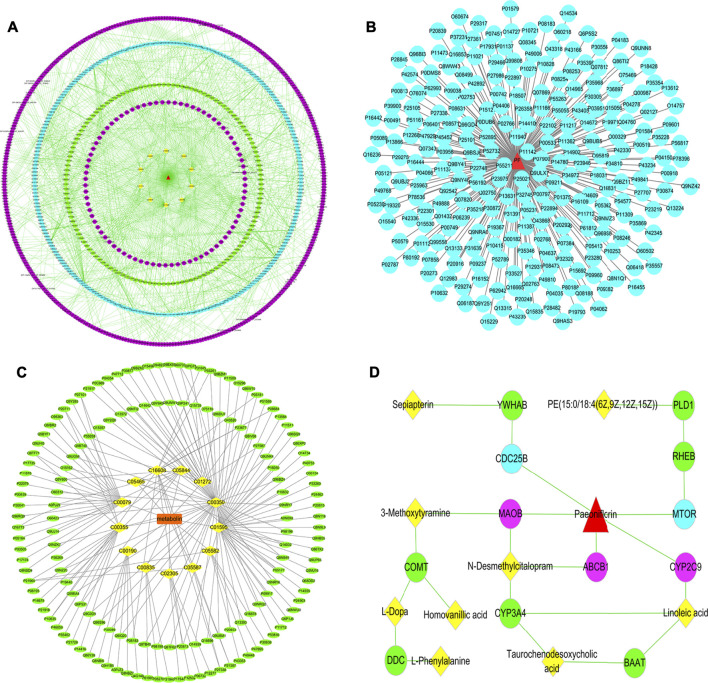
**(A)** The “specific metabolite-target-component” interactive network; **(B)** The “PF-target” interactive network; **(C)** The “potential metabolite-target” interactive network. The blue dots represent the protein targets of PF. The green dots represent the protein targets of potential metabolites. The purple dots represent interacting proteins. The yellow squares represent potential metabolites. **(D)** The pivotal “specific metabolite-target-component” interactive network of PF in the treatment of cholestatic liver injury. The red triangles represent PF. The blue dots represent the protein targets of PF. The green dots represent the protein targets of potential metabolites. The yellow squares represent potential metabolites. The purple dots represent the direct targets of PF in regulating potential metabolites.

To systematically clarify the complex relationship between PF and potential biomarkers, the targets of PF regulating specific metabolites were analyzed by network pharmacology. MBROLE 2.0 was performed for collecting specific metabolite-related targets. As shown in Figures 6B,C, we identified 166 targets corresponding to 13 potential metabolites and 248 protein PF targets. Among them, ten target-specific metabolites, 244 PF targets, 150 specific metabolite targets, and 827 interacting proteins participated in the construction of the “potential metabolite-target-component” interaction network together (Figure 6A).

Herein, PF directly regulated the metabolite linoleic acid, N-desmethylcitalopram, 3-methoxytyramine by acting on the cytochrome P450 2C9 (CYP2C9), ATP-dependent translocase ABCB1 (ABCB1), Amine oxidase [flavin-containing] B (MAOB) target, and indirectly regulated the potential metabolites of PE [15:0/18:4 (6Z,9Z,12Z,15Z)] and sepiapterin by acting on serine/threonine-protein kinase mTOR (MTOR) and M-phase inducer phosphatase 2 (CDC25B) target (Figure 6D).

### Effect of PF on the Expression of Key Targets Related to Potential Biomarkers

**FIGURE 7:**
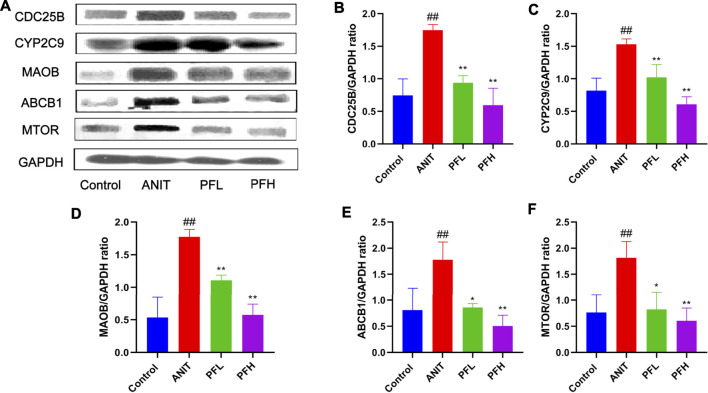
Effect of PF on the expression of CYP2C9, ABCB1, MAOB, CDC25B, and MTOR in the treatment of cholestatic liver injury detected by western blotting. **(A)** Western blotting images of CYP2C9, ABCB1, MAOB, CDC25B, and MTOR; **(B)** Relative protein expression of CDC25B; **(C)** Relative protein expression of CYP2C9; **(D)** Relative protein expression of MAOB; **(E)** Relative protein expression of ABCB1; **(F)** Relative protein expression of MTOR. The data are expressed as the mean ± SD, *n* = 3. *##* *p <* 0.01, *#* *p <* 0.05 compared with the control group, ** *p <* 0.01, * *p <* 0.05 compared with ANIT group.

**FIGURE 8:**
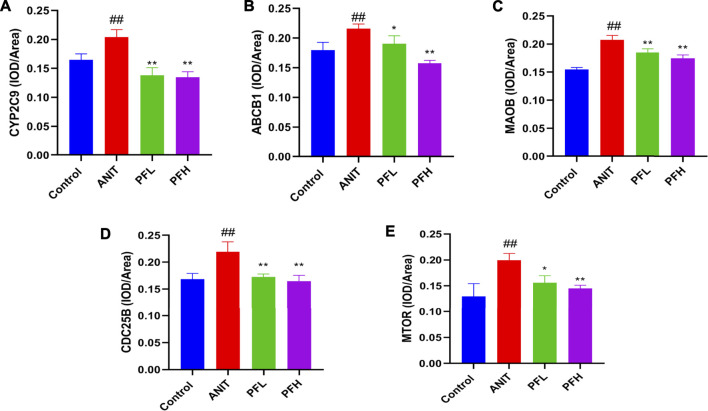
Effect of PF on the expression of CYP2C9, ABCB1, MAOB, CDC25B, and MTOR in the treatment of cholestatic liver injury. **(A)** The expression of CYP2C9 in the liver tissue; **(B)** The expression of ABCB1 in the liver tissue; **(C)** The expression of MAOB in the liver tissue; **(D)** The expression of CDC25B in the liver tissue; **(E)** The expression of MTOR in the liver tissue. The data are expressed as the mean ± SD, *n* = 3. ^*##*^ *p <* 0.01, ^*#*^ *p <* 0.05 compared with the control group, ^**^ *p <* 0.01, ^*^ *p <* 0.05 compared with ANIT group.

To verify the authenticity of network pharmacological prediction, we used immunohistochemistry and western blotting to verify the targets of PF directly or indirectly regulating potential metabolites. The results indicated the expression of CYP2C9, ABCB1, MAOB, MTOR, and CDC25B were significantly increased in the liver tissues of rats in the ANIT group, while the PF significantly reduced the expression of these proteins (*p <* 0.01 or *p <* 0.05). The results of immunohistochemistry were consistent with those of western blotting. (Figures 7, 8, Supplementary Figure S3).

## Discussion

Cholestasis is the intrahepatic accumulation of toxic bile acids that occurs in several liver diseases, which were caused by various factors, such as pregnancy, drugs, alcohol, etc (Chatterjee and Annaert, 2018; Ovadia et al., 2021; Takeuchi et al., 2021). The occurrence of cholestatic liver diseases is very rare, with an annual incidence of between 0.3/100000 and 5.8/100000 (Wagner and Fickert, 2020). The enterohepatic circulation of bile acid describes the physiological interaction between intestine and liver and is of great significance for the maintenance of health. Feces play an important role in the hepato-intestinal circulation. Secondary bile acids in feces initiate the metabolism of bile acids in the intestinal microflora. (Ocvirk and O'Keefe, 2021). Previous studies have shown that fecal microbiota functional diversity is significantly reduced in patients with liver disease compared to healthy controls, and both intestinal microbiome and fecal transplantation techniques have demonstrated the critical role of feces in bile acid metabolism-related diseases (Out et al., 2015; Wei et al., 2013). Paeoniflorin is the main component of *Paeonia lactiflora* Pall.. Studies have shown that PF can effectively relieve cholestasis (Zhao et al., 2017), however, its effect on cholestatic liver injury has not been completely clear in the metabolic environment. Therefore, the metabolic study of feces can further understand the mechanism of PF in improving cholestatic liver injury. Metabolomics has a comprehensive understanding of the mechanism of multi-target and multi-pathway of traditional Chinese medicine in protecting liver, and screening biomarkers and metabolic pathways to protect liver, but the related targets of regulating biomarkers are not clear. Network pharmacology can analyze the interaction between targets corresponding to chemical components and diseases, and reveal the molecular mechanism of drug therapy (Huo et al., 2018; Wu et al., 2020). The combination of metabolomics and network pharmacology provides a more comprehensive and in-depth explanation of the role of PF in liver protection by regulating biomarkers through which targets.

In this study, PF significantly improved serum biochemical indexes and alleviated liver tissue injury. In addition, the metabolomics method was used to obtain the metabolomics characteristic map of paeoniflorin anti-cholestasis liver injury and the interaction network of metabolic differentiators. The results showed that PF restored 13 potential biomarkers, including inositol 1,3,4,5-Tetrakisphosphate, sepiapterin, N-desmethylcitalopram, UDP-D-Xylose, taurochenodesoxycholic acid, L-dopa, 3-methoxytyramine, homovanillic acid, L-phenylalanine, linoleic acid, 5-L-glutamyl-taurine, PE [15:0/18:4 (6Z,9Z,12Z,15Z)], and phosphocreatine to normal levels, which were mainly related to amino acid metabolism, bile acid metabolism, and inflammation-related metabolism. Metabolic pathway analysis showed that paeoniflorin had a protective effect on cholestatic liver injury mainly by regulating linoleic acid metabolism, phenylalanine, tyrosine and tryptophan biosynthesis, phenylalanine metabolism, tyrosine metabolism, phosphatidylinositol signaling system, inositol phosphate metabolism, primary bile acid biosynthesis, glycosylphosphatidylinositol (GPI)-anchor biosynthesis. The above metabolic pathways were mainly related to amino acid metabolic pathway, bile acid metabolic pathway and inflammation-related pathway. Amino acid metabolism exists in every cell of the organism, and the occurrence of disease and health status are directly or indirectly related to amino acids. Studies have shown that severe damage to liver cells can cause amino acid metabolism disorder (Loza-Valdes et al., 2021). By studying the significantly changed amino acids, we can further clarify the mechanism of PF in the treatment of cholestasis liver injury. Clinically, the severity of hepatic encephalopathy and liver disease is usually predicted according to the ratio of branched chain amino acids to aromatic amino acids. In this study, PF significantly improved the metabolic pathways of tyrosine and phenylalanine. Primary bile acid metabolism also plays an important role in the regulation of cholestatic liver injury. Primary bile acid flows into the intestine with bile and forms secondary bile acid under the action of intestinal flora, which is reabsorbed through the intestinal wall and returned to the liver, and then discharged into the intestine through the biliary tract to form hepatointestinal circulation (Kummen and Hov, 2019). This study found that PF significantly improved the level of taurodeoxycholic acid, suggesting that PF may alleviate cholestatic liver injury by regulating bile acid-related metabolism. In addition, bile acid can also be used as an inflammatory stimulator to stimulate the production of many inflammatory mediators, thus promoting the inflammatory response of the liver. Studies have shown that linoleic acid metabolism and phosphatidylinositol-related metabolism are involved in a variety of inflammatory signal transduction (Owusu Obeng et al., 2020; Wang et al., 2021). The result showed that PF played a protective role in cholestatic liver injury by regulating inflammation-related pathways.

To have a deeper understanding of how PF regulates potential biomarkers, we used network pharmacology method to analyze the targets of PF and potential biomarkers, and construct the interactive network of “potential biomarkers-targets-components”. The results showed that PF regulated five metabolites including linoleic acid, N-desmethylcitalopram, 3-methoxytyramine, PE [15:0/18:4 (6Z,9Z,12Z,15Z)] and sepiapterin through CDC25B, CYP2C9, MAOB, mTOR, and ABCB1. CDC25B, an important member of the CDC25 family, is a threonine/tyrosine bispecific protein phosphatase. It is an important factor in regulating the activity of CDK-cyclin complex. When CyclinA and CDK combine, CDK molecule exposes threonine and tyrosine at positions 14 and 15. Kinase Weel can phosphorylate threonine and tyrosine at positions 14 and 15, which plays a key role in regulating the ability of hepatocyte regeneration (Shen, 2007; Wang et al., 2002). CYP450 enzyme system is the largest superfamily of drug metabolic enzymes, which can catalyze the oxidation of drugs. CYP2C9 is the main member of the CYP2C subfamily, which has a high content in the liver, accounting for 20% of the weight of liver microsomal P450, and mediates a variety of drug metabolism. Its structural and functional characteristics and its role in metabolism have been paid more and more attention. It has been reported that PF inhibits the activity of CYP2C9 enzyme (Fan et al., 2019). MAOB is a kind of monoamine oxidase, which is mainly distributed in the mitochondria of liver, kidney, and other tissues. Hydrogen peroxide produced in the activated state will lead to oxidative stress, damage the function of mitochondria, and then affect its energy metabolism. Oxidative stress and inflammation are inseparable, so inhibition of MAOB can significantly inhibit inflammation (Qian et al., 2021). MTOR, the target protein of rapamycin, is a member of the protein family of phosphatidylinositol 3 kinase related enzymes. A variety of inflammatory factors mediate the inflammatory response of the liver through mTOR-mediated signaling pathways, which change the structure of the liver. Studies have shown that inhibiting the activation of PI3K/Akt/mTOR pathway can significantly reduce the production of inflammatory mediators, thus alleviating the inflammatory response (Salti et al., 2020; Huiming et al., 2021). ABCB1 is the first ABC transporter subtype discovered, also known as multi-drug resistance protein 1 (MDR1) or P-gp glycoprotein (P-gp), which is mainly expressed in liver. ABCB1 absorbs and transfers out of the cells the exogenous substances that enter the body and the harmful toxins produced by the body’s metabolism. The transporter MDR1 located at the top of the bile capillary is the determinant of bile secretion and composition, and its main function is to transport hydrophobic compounds into the bile (Boyer and Soroka, 2021). In addition, some studies have shown that MDR1 promoted the excretion of bilirubin and bile acid, and accelerated the efflux of hepatotoxic substances, so as to dispel jaundice, cholagogic and detoxification, thereby regulating bile acid metabolism (Tang et al., 2016; Jetter and Kullak-Ublick, 2020). Other studies have shown that MDR1 was involved in inflammation, and the lack of MDR1 led to mitochondrial dysfunction and the increase of mitochondrial reactive oxygen species, which promoted the development of colitis (Ho et al., 2018). Therefore, MDR1 is an important target for alleviating inflammation and regulating bile acid metabolism.

To confirm the key targets selected above and better reveal the mechanism of PF regulating biomarkers, we used immunohistochemistry and western blotting to verify the above five key targets. The results showed that CDC25B, CYP2C9, MAOB, mTOR, and ABCB1 increased significantly in the ANIT group, while the expression of these proteins decreased significantly in the PF group. Taken together, these five key targets may be a favorable explanation for the effects of PF on the above-mentioned metabolic pathways. The above findings suggest that PF regulates bile acid metabolism mainly by regulating the synthesis and efflux of bile acid and bilirubin. In addition, PF can also alleviate cholestatic liver injury by reducing inflammation. According to the result above, PF could reverse the metabolic disorder caused by cholestatic liver injury induced by ANIT and effectively curb the rapid development of cholestatic liver injury, which shows that PF can treat cholestatic liver injury by multi-target and multi-pathway.

## Conclusion

In conclusion, our study systematically explored the molecular mechanism of PF for the treatment of cholestatic liver injury by combining metabolomics, network pharmacology and molecular biology methods. The results indicated that PF, especially the high dose of PF, had a protective effect on cholestatic liver injury by regulating amino acid metabolism, bile acid metabolism and inflammation, which reflected the multiple pathways and multiple targets of PF in relieving cholestatic liver injury. These findings suggest that PF may be a promising agent candidate for the treatment of cholestasis liver injury, and the selected targets can be used as potential drug targets for the diagnosis or treatment of cholestasis.

## Data Availability

The original contributions presented in the study are included in the article/Supplementary Material, further inquiries can be directed to the corresponding author.

## References

[B1] Blesl A. Stadlbauer V. (2021). The Gut-Liver Axis in Cholestatic Liver Diseases. Nutrients 13 (3). 1018. 10.3390/nu13031018 33801133 PMC8004151

[B2] Boyer J. L. Soroka C. J. (2021). Bile Formation and Secretion: An Update. J. Hepatol. 75 (1), 190–201. 10.1016/j.jhep.2021.02.011 33617926

[B3] Chatterjee S. Annaert P. (2018). Drug-induced Cholestasis: Mechanisms, Models, and Markers. Curr. Drug Metab. 19 (10), 808–818. 10.2174/1389200219666180427165035 29708070

[B4] Chen H. L. Wu S. H. Hsu S. H. Liou B. Y. Chen H. L. Chang M. H. (2018). Jaundice Revisited: Recent Advances in the Diagnosis and Treatment of Inherited Cholestatic Liver Diseases. J. Biomed. Sci. 25 (1), 75. 10.1186/s12929-018-0475-8 30367658 PMC6203212

[B5] Chen X. Zhang J. Wang R. Liu H. Bao C. Wu S. (2020). UPLC-Q-TOF/MS-Based Serum and Urine Metabonomics Study on the Ameliorative Effects of Palmatine on Helicobacter Pylori-Induced Chronic Atrophic Gastritis. Front. Pharmacol. 11, 586954. 10.3389/fphar.2020.586954 33041831 PMC7522567

[B6] Chen Z. Zhu Y. Zhao Y. Ma X. Niu M. Wang J. (2016). Serum Metabolomic Profiling in a Rat Model Reveals Protective Function of Paeoniflorin against ANIT Induced Cholestasis. Phytother Res. 30 (4), 654–662. 10.1002/ptr.5575 26806614

[B7] Cheng J. Chen M. Wan H. Q. Chen X. Q. Li C. F. Zhu J. X. (2021). Paeoniflorin Exerts Antidepressant-like Effects through Enhancing Neuronal FGF-2 by Microglial Inactivation. J. Ethnopharmacol 274, 114046. 10.1016/j.jep.2021.114046 33753146

[B8] Fan Z. Ma S. Xu A. Wang Y. (2019). Proluvial Fan Landscape. Herald Med. 38 (04), 486. 10.1007/978-981-13-2538-0_1945

[B9] Ho G. T. Aird R. E. Liu B. Boyapati R. K. Kennedy N. A. Dorward D. A. (2018). MDR1 Deficiency Impairs Mitochondrial Homeostasis and Promotes Intestinal Inflammation. Mucosal Immunol. 11 (1), 120–130. 10.1038/mi.2017.31 28401939 PMC5510721

[B10] Huiming G. Jianyuan G. Yuan Q. Huawei W. Jiang L. Qingyan P. (2021). miR-125b-5p Inhibits Cell Proliferation by Targeting ASCT2 and Regulating the PI3K/AKT/mTOR Pathway in an LPS-Induced Intestinal Mucosa Cell Injury Model. Exp. Ther. Med. 22 (2), 838. 10.3892/etm.2021.10270 34149884 PMC8210225

[B11] Huo X. K. Liu J. Yu Z. L. Wang Y. F. Wang C. Tian X. G. (2018). Alisma Orientale Extract Exerts the Reversing Cholestasis Effect by Activation of Farnesoid X Receptor. Phytomedicine 42, 34–42. 10.1016/j.phymed.2018.03.017 29655695

[B12] Jansen P. L. Ghallab A. Vartak N. Reif R. Schaap F. G. Hampe J. (2017). The Ascending Pathophysiology of Cholestatic Liver Disease. Hepatology 65 (2), 722–738. 10.1002/hep.28965 27981592

[B13] Jetter A. Kullak-Ublick G. A. (2020). Drugs and Hepatic Transporters: A Review. Pharmacol. Res. 154, 104234. 10.1016/j.phrs.2019.04.018 31004787

[B14] Kong X. Leng D. Liang G. Zheng H. Wang Q. Shen Y. (2018). Paeoniflorin Augments Systemic Candida Albicans Infection through Inhibiting Th1 and Th17 Cell Expression in a Mouse Model. Int. Immunopharmacol 60, 76–83. 10.1016/j.intimp.2018.03.001 29705532

[B15] Kowdley K. V. Luketic V. Chapman R. Hirschfield G. M. Poupon R. Schramm C. (2018). A Randomized Trial of Obeticholic Acid Monotherapy in Patients with Primary Biliary Cholangitis. Hepatology 67 (5), 1890–1902. 10.1002/hep.29569 29023915 PMC5947631

[B16] Kummen M. Hov J. R. (2019). The Gut Microbial Influence on Cholestatic Liver Disease. Liver Int. 39 (7), 1186–1196. 10.1111/liv.14153 31125502

[B17] Loza-Valdes A. Mayer A. E. Kassouf T. Trujillo-Viera J. Schmitz W. Dziaczkowski F. (2021). A Phosphoproteomic Approach Reveals that PKD3 Controls PKA-Mediated Glucose and Tyrosine Metabolism. Life Sci. Alliance 4 (8). 10.26508/lsa.202000863 PMC8321662 34145024

[B18] Lyu M. Zhou Z. Wang X. Lv H. Wang M. Pan G. (2018). Network Pharmacology-Guided Development of a Novel Integrative Regimen to Prevent Acute Graft-vs.-Host Disease. Front. Pharmacol. 9, 1440. 10.3389/fphar.2018.01440 30618740 PMC6300759

[B19] Marrero I. Maricic I. Feldstein A. E. Loomba R. Schnabl B. Rivera-Nieves J. (2018). Complex Network of NKT Cell Subsets Controls Immune Homeostasis in Liver and Gut. Front. Immunol. 9, 2082. 10.3389/fimmu.2018.02082 30254647 PMC6141878

[B20] Milosevic I. Vujovic A. Barac A. Djelic M. Korac M. Radovanovic Spurnic A. (2019). Gut-Liver Axis, Gut Microbiota, and its Modulation in the Management of Liver Diseases: A Review of the Literature. Int. J. Mol. Sci. 20 (2), 395. 10.3390/ijms20020395 PMC6358912 30658519

[B21] Ocvirk S. O'Keefe S. J. D. (2021). Dietary Fat, Bile Acid Metabolism and Colorectal Cancer. Semin. Cancer Biol. 73, 347–355. 10.1016/j.semcancer.2020.10.003 33069873

[B22] Out C. Patankar J. V. Doktorova M. Boesjes M. Bos T. de Boer S. (2015). Gut Microbiota Inhibit Asbt-dependent Intestinal Bile Acid Reabsorption *via* Gata4. J. Hepatol. 63 (3), 697–704. 10.1016/j.jhep.2015.04.030 26022694 PMC5293168

[B23] Ovadia C. Sajous J. Seed P. Patel K. Williamson N. Attilakos G. (2021). Ursodeoxycholic Acid in Intrahepatic Cholestasis of Pregnancy: a Systematic Review and Individual Participant Data Meta-Analysis. *The Lancet* . Gastroenterol. Hepatol. 6 (7), 547–558. 10.1016/s2468-1253(21)00074-1 PMC8192305 33915090

[B24] Owusu Obeng E. Rusciano I. Marvi M. V. Fazio A. Ratti S. Follo M. Y. (2020). Phosphoinositide-Dependent Signaling in Cancer: A Focus on Phospholipase C Isozymes. Int. J. Mol. Sci. 21 (7). 2581, 10.3390/ijms21072581 PMC7177890 32276377

[B25] Qian L. Li J. Z. Sun X. Chen J. B. Dai Y. Huang Q. X. (2021). Safinamide Prevents Lipopolysaccharide (LPS)-induced Inflammation in Macrophages by Suppressing TLR4/NF-Κb Signaling. Int. Immunopharmacol 96, 107712. 10.1016/j.intimp.2021.107712 34162132

[B26] Salti T. Khazim K. Haddad R. Campisi-Pinto S. Bar-Sela G. Cohen I. (2020). Glucose Induces IL-1α-Dependent Inflammation and Extracellular Matrix Proteins Expression and Deposition in Renal Tubular Epithelial Cells in Diabetic Kidney Disease. Front. Immunol. 11, 1270. 10.3389/fimmu.2020.01270 32733443 PMC7358427

[B27] Shen L. (2007). Role of P38MAPK in Cell Proliferation and Apoptosis in Human Hepatocarcinogenesis (Doctor). Kunming: Kunming Medical College.

[B28] Takeuchi M. Vidigal P. T. Guerra M. T. Hundt M. A. Robert M. E. Olave-Martinez M. (2021). Neutrophils Interact with Cholangiocytes to Cause Cholestatic Changes in Alcoholic Hepatitis. Gut. 70 (2), 342–356. 10.1136/gutjnl-2020-322540 33214166 PMC7906004

[B29] Tang X. Yang Q. Yang F. Gong J. Han H. Yang L. (2016). Target Profiling Analyses of Bile Acids in the Evaluation of Hepatoprotective Effect of Gentiopicroside on ANIT-Induced Cholestatic Liver Injury in Mice. J. Ethnopharmacol 194, 63–71. 10.1016/j.jep.2016.08.049 27582267

[B30] Tian F. Gu L. Si A. Yao Q. Zhang X. Zhao J. (2015). Metabolomic Study on the Faecal Extracts of Atherosclerosis Mice and its Application in a Traditional Chinese Medicine. J. Chromatogr. B Analyt Technol. Biomed. Life Sci. 1007, 140–148. 10.1016/j.jchromb.2015.10.016 26596842

[B31] Tu J. Guo Y. Hong W. Fang Y. Han D. Zhang P. (2019). The Regulatory Effects of Paeoniflorin and its Derivative Paeoniflorin-6,-O-Benzene Sulfonate CP-25 on Inflammation and Immune Diseases. Front. Pharmacol. 10, 57. 10.3389/fphar.2019.00057 30804784 PMC6370653

[B32] Wagner M. Fickert P. (2020). Drug Therapies for Chronic Cholestatic Liver Diseases. Annu. Rev. Pharmacol. Toxicol. 60, 503–527. 10.1146/annurev-pharmtox-010818-021059 31506007

[B33] Wang J. Xu D. Shen L. Zhou J. Lv X. Ma H. (2021). Anti-inflammatory and Analgesic Actions of Bufotenine through Inhibiting Lipid Metabolism Pathway. Biomed. Pharmacother. 140, 111749. 10.1016/j.biopha.2021.111749 34058437

[B34] Wang X. Krupczak-Hollis K. Tan Y. Dennewitz M. B. Adami G. R. Costa R. H. (2002). Increased Hepatic Forkhead Box M1B (FoxM1B) Levels in Old-Aged Mice Stimulated Liver Regeneration through Diminished p27Kip1 Protein Levels and Increased Cdc25B Expression. J. Biol. Chem. 277 (46), 44310–44316. 10.1074/jbc.M207510200 12221098

[B35] Wang X. Sun H. Zhang A. Sun W. Wang P. Wang Z. (2011). Potential Role of Metabolomics Apporoaches in the Area of Traditional Chinese Medicine: as Pillars of the Bridge between Chinese and Western Medicine. J. Pharm. Biomed. Anal. 55 (5), 859–868. 10.1016/j.jpba.2011.01.042 21353755

[B36] Wei S. Ma X. Niu M. Wang R. Yang T. Wang D. (2020). Mechanism of Paeoniflorin in the Treatment of Bile Duct Ligation-Induced Cholestatic Liver Injury Using Integrated Metabolomics and Network Pharmacology. Front. Pharmacol. 11, 586806. 10.3389/fphar.2020.586806 33192530 PMC7641625

[B37] Wei X. Yan X. Zou D. Yang Z. Wang X. Liu W. (2013). Abnormal Fecal Microbiota Community and Functions in Patients with Hepatitis B Liver Cirrhosis as Revealed by a Metagenomic Approach. BMC Gastroenterol. 13, 175. 10.1186/1471-230x-13-175 24369878 PMC3878425

[B38] Wu J. Fang S. Li W. Li Y. Li Y. Wang T. (2020). Metabolomics Research on the Hepatoprotective Effect of Cultured bear Bile Powder in α-naphthylisothiocyanate-induced Cholestatic Mice. J. Chromatogr. B Analyt Technol. Biomed. Life Sci. 1153, 122269. 10.1016/j.jchromb.2020.122269 32739790

[B39] Xi-lan W. L.-j. Y. X.-c. K. J.-q. D. S.-y. H. C. X. Y.-h. T. (2021). Research Progress in Treatment of Chemical Liver Injury with Chinese Medicine Based on Metabolomics. Chin. J. Exp. Traditional Med. Formulae 27, 203–215. 10.13422/j.cnki.syfjx.20210716

[B40] Xiong A. Yang F. Fang L. Yang L. He Y. Wan Y. J. (2014). Metabolomic and Genomic Evidence for Compromised Bile Acid Homeostasis by Senecionine, a Hepatotoxic Pyrrolizidine Alkaloid. Chem. Res. Toxicol. 27 (5), 775–786. 10.1021/tx400451q 24641316 PMC7229698

[B41] Xu T. Pi Z. Liu S. Song F. Liu Z. (2017). Chemical Profiling Combined with "Omics" Technologies (CP-Omics): a Strategy to Understand the Compatibility Mechanisms and Simplify Herb Formulas in Traditional Chinese Medicines. Phytochem. Anal. 28 (5), 381–391. 10.1002/pca.2685 28387961

[B42] Zhang J. W. Li L. X. Wu W. Z. Pan T. J. Yang Z. S. Yang Y. K. (2018). Anti-Tumor Effects of Paeoniflorin on Epithelial-To-Mesenchymal Transition in Human Colorectal Cancer Cells. Med. Sci. Monit. 24, 6405–6413. 10.12659/msm.912227 30208371 PMC6146785

[B43] Zhang R. Zhu X. Bai H. Ning K. (2019). Network Pharmacology Databases for Traditional Chinese Medicine: Review and Assessment. Front. Pharmacol. 10, 123. 10.3389/fphar.2019.00123 30846939 PMC6393382

[B44] Zhao Y. He X. Ma X. Wen J. Li P. Wang J. (2017). Paeoniflorin Ameliorates Cholestasis *via* Regulating Hepatic Transporters and Suppressing Inflammation in ANIT-Fed Rats. Biomed. Pharmacother. 89, 61–68. 10.1016/j.biopha.2017.02.025 28214689

[B45] Zhao Y. Zhou G. Wang J. Jia L. Zhang P. Li R. (2013). Paeoniflorin Protects against ANIT-Induced Cholestasis by Ameliorating Oxidative Stress in Rats. Food Chem. Toxicol. 58, 242–248. 10.1016/j.fct.2013.04.030 23623840

[B46] Zhu G. Feng F. (2019). UPLC-MS-based Metabonomic Analysis of Intervention Effects of Da-Huang-Xiao-Shi Decoction on ANIT-Induced Cholestasis. J. Ethnopharmacol 238, 111860. 10.1016/j.jep.2019.111860 30965080

