# Supplementary figures and images for: Mechanism of Paeoniflorin on ANIT-Induced Cholestatic Liver Injury Using Integrated Metabolomics and Network Pharmacology
Source: Front Pharmacol. 2021 Aug 30;12:737630. doi: 10.3389/fphar.2021.737630 (PMC8435635; doi:10.3389/fphar.2021.737630)

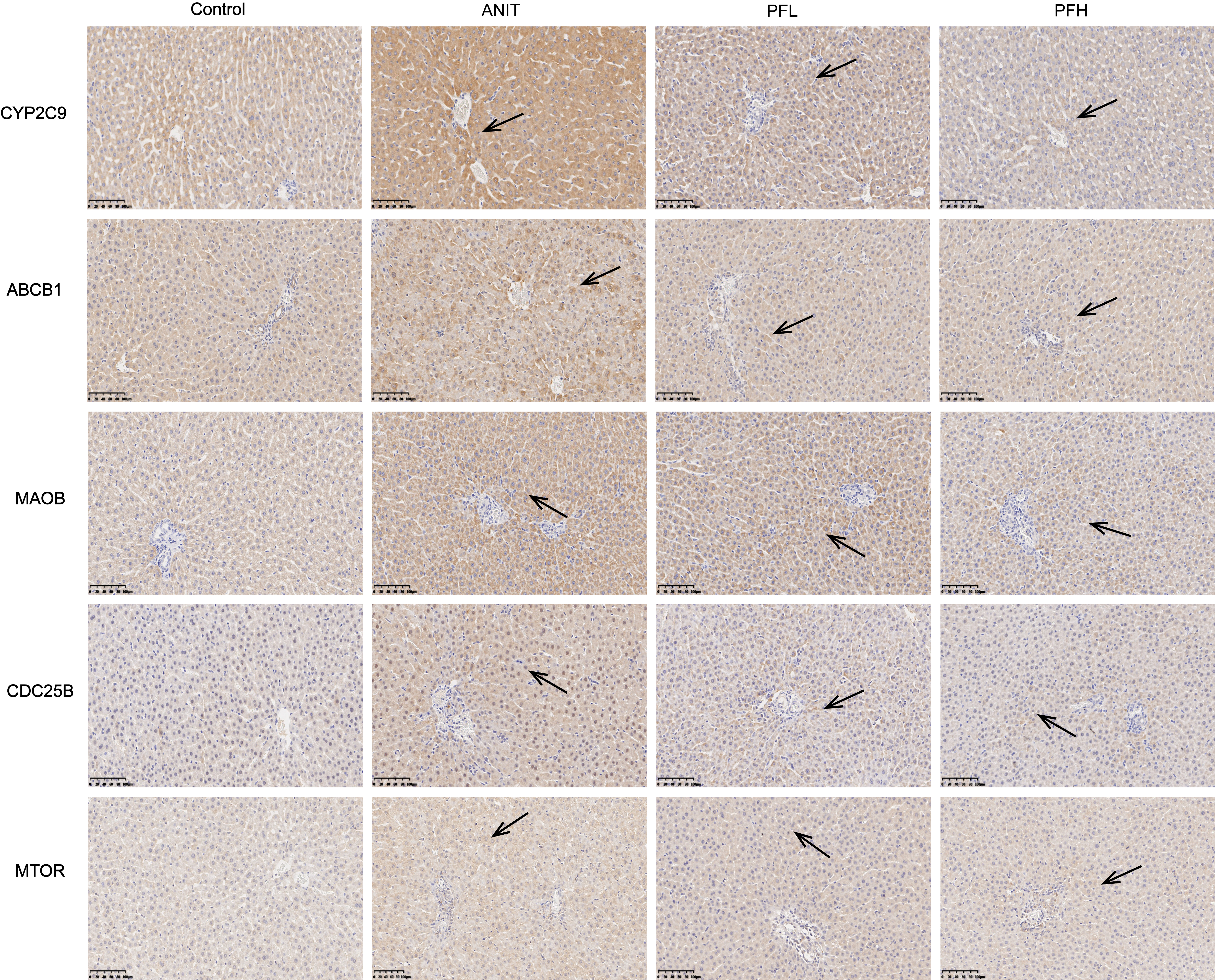

Supplement: Supplementary file 2 [file Image3.JPEG]

Control:


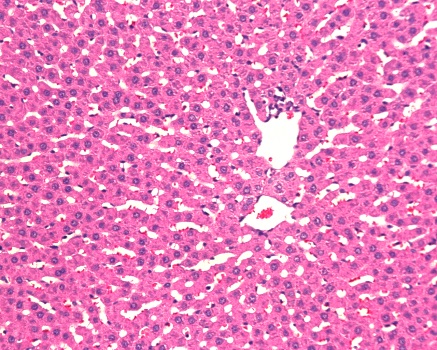

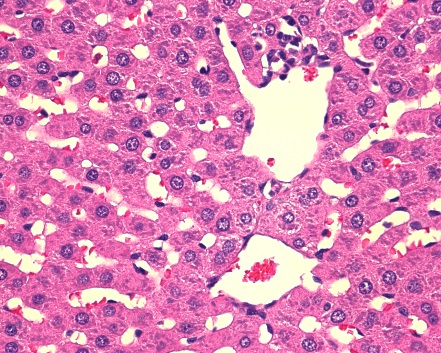


200× 400×

ANIT:


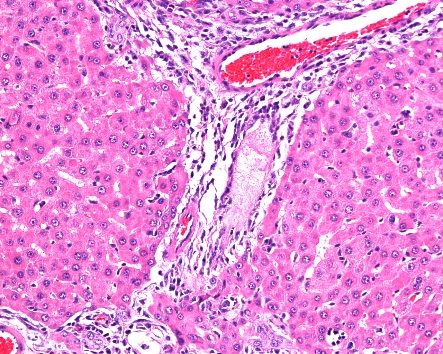

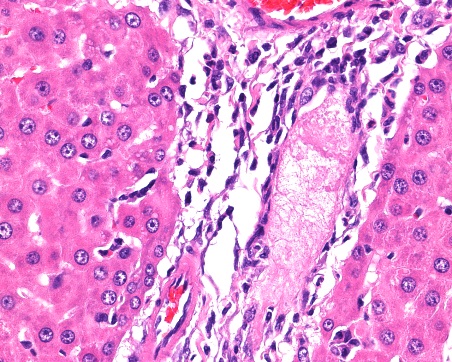


200× 400×

UDCA:


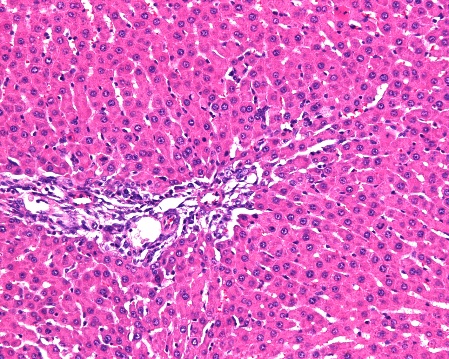

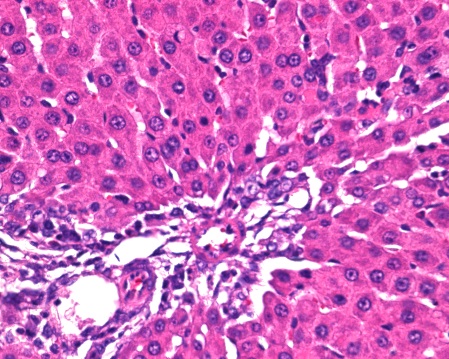


200× 400×

PFL:


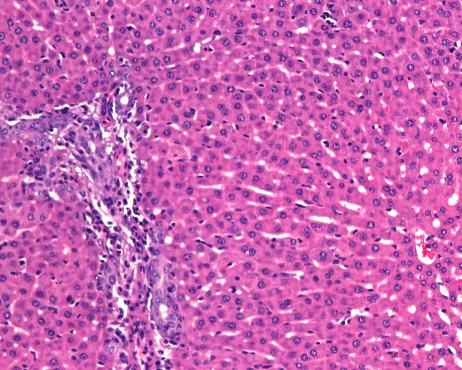

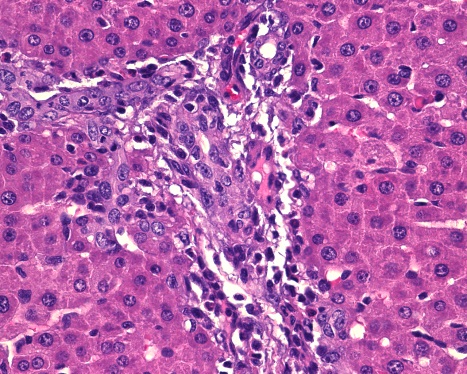


200× 400×

PFH:


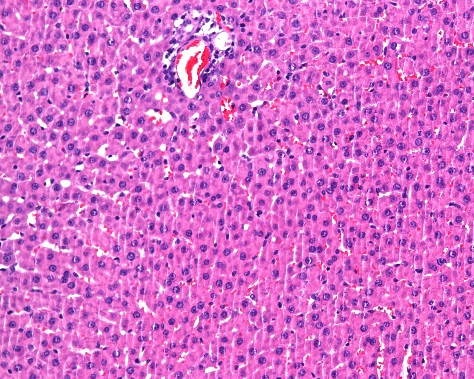

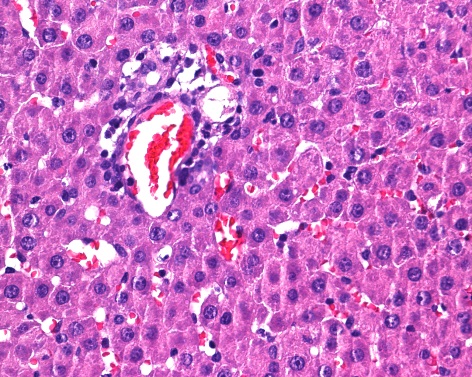


200× 400×

Supplement: Supplementary file 4 [file DataSheet1.ZIP › Figure 3-Original data of liver histopathology.docx]

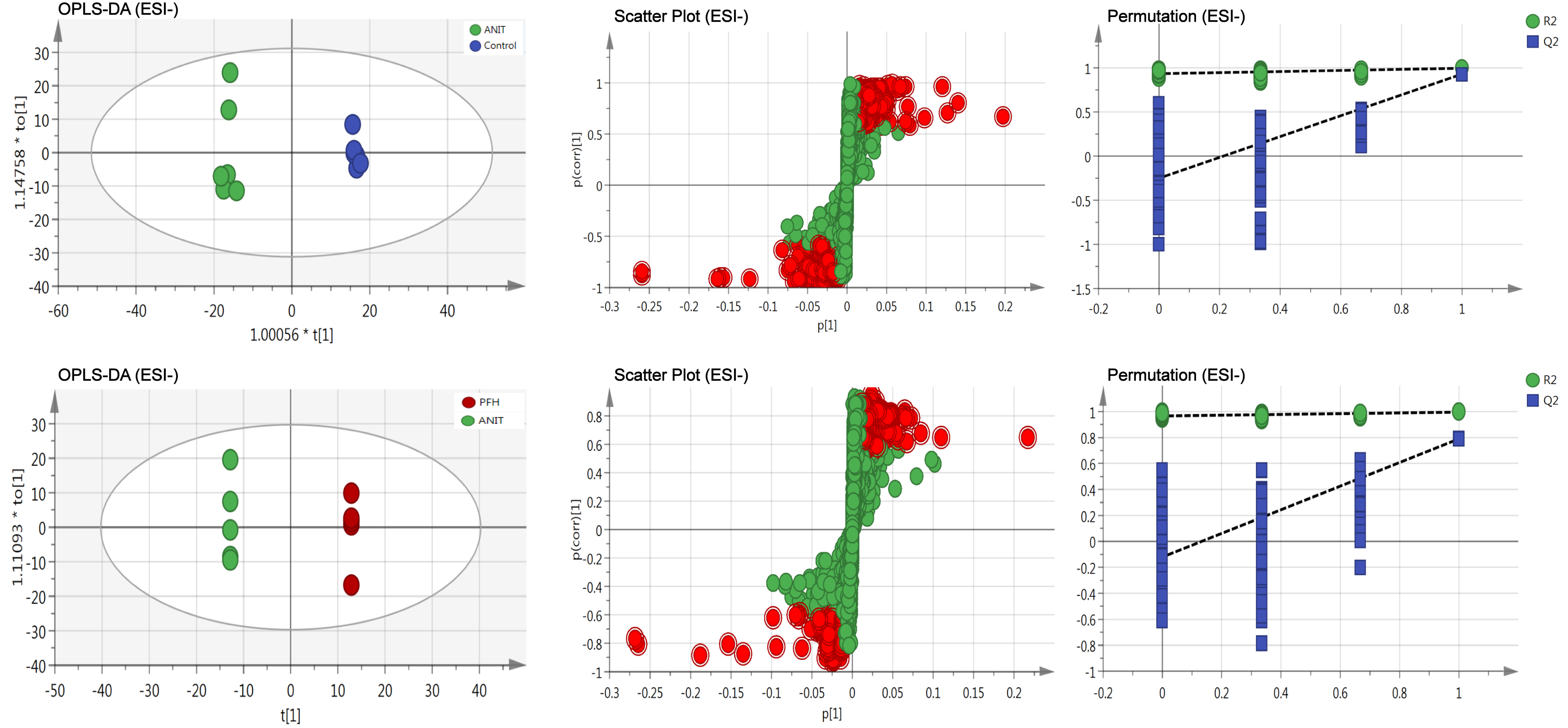

Supplement: Supplementary file 5 [file Image2.JPEG]

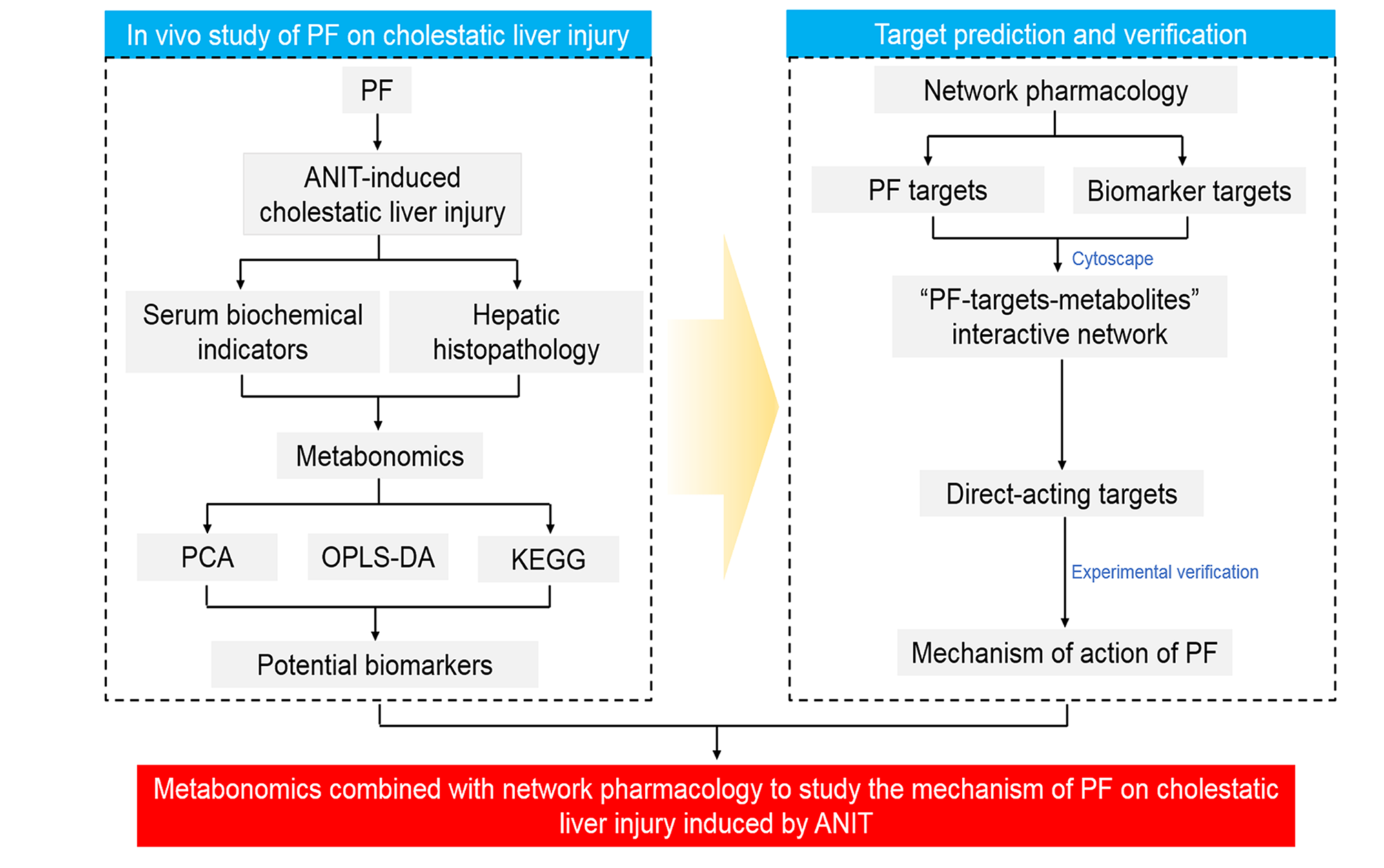

Supplement: Supplementary file 7 [file Image1.TIF]
