# Supplementary material for: Mechanism of Paeoniflorin on ANIT-Induced Cholestatic Liver Injury Using Integrated Metabolomics and Network Pharmacology
Source: Front Pharmacol. 2021 Aug 30;12:737630. doi: 10.3389/fphar.2021.737630 (PMC8435635; doi:10.3389/fphar.2021.737630)
Supplement: Supplementary file 8 [file DataSheet2.ZIP › Figure 9-Original data of immunohistochemical result.docx]

**CYP2C9:**

**Control: ANIT:**

**
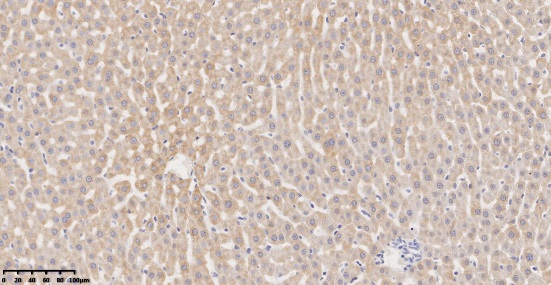

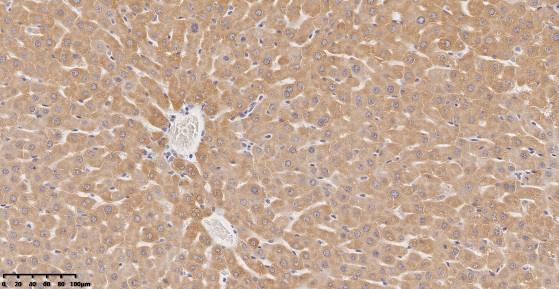
**

**PFL: PFH:**

**
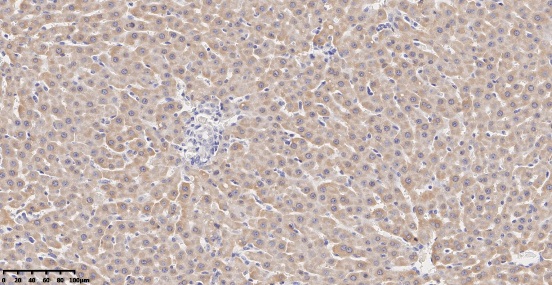

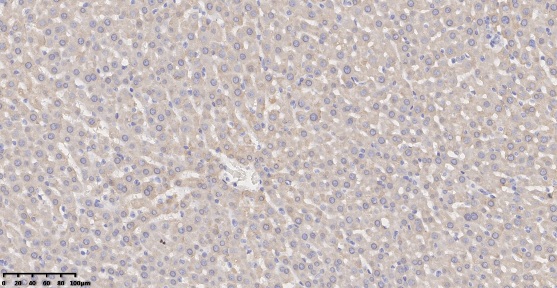
**

**ABCB1:**

**Control: ANIT:**

**
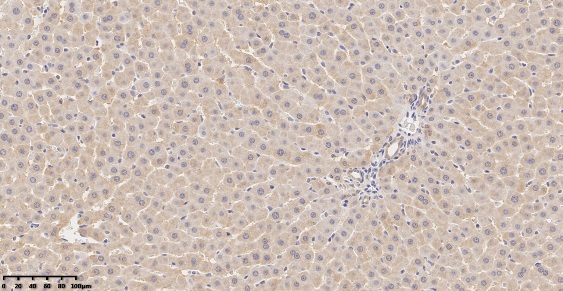

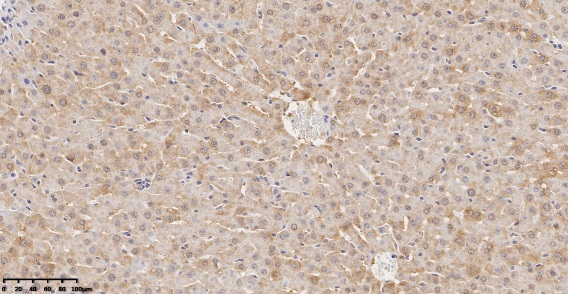
**

**PFL: PFH:**

**
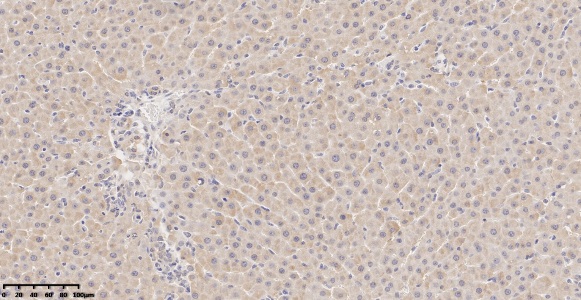

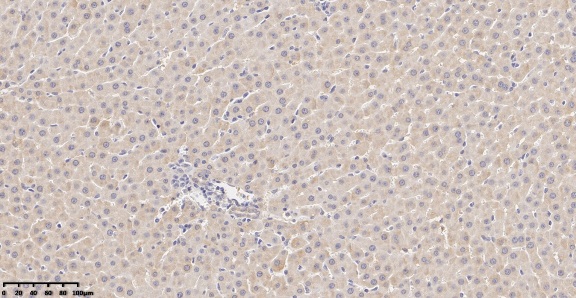
**

**MAOB:**

**Control: ANIT:**

**
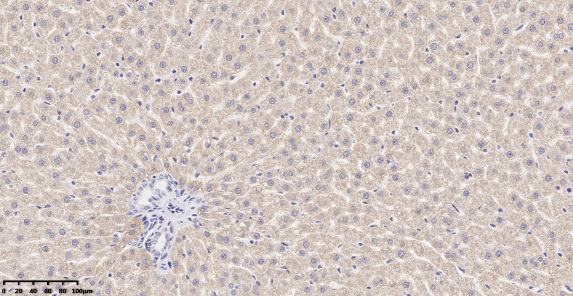

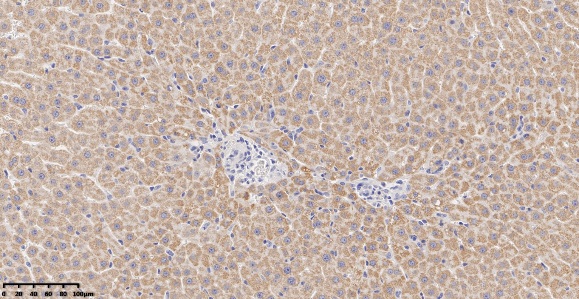
**

**PFL: PFH:**

**
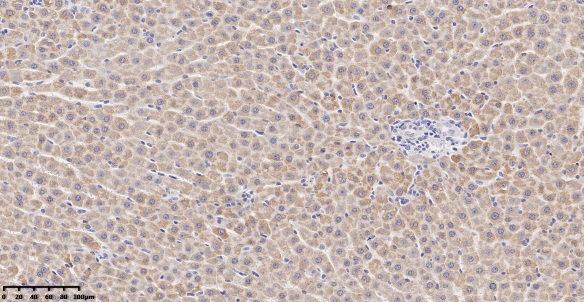

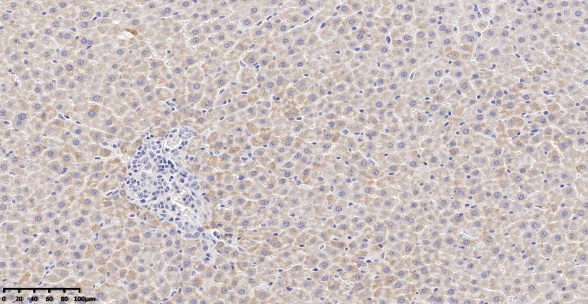
**

**CDC25B:**

**Control: ANIT:**

**
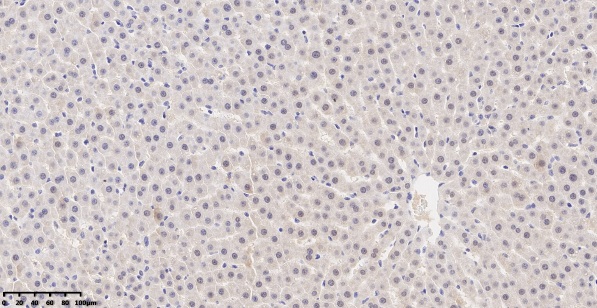

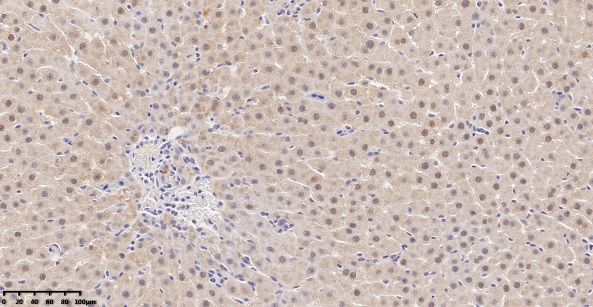
**

**PFL: PFH:**

**
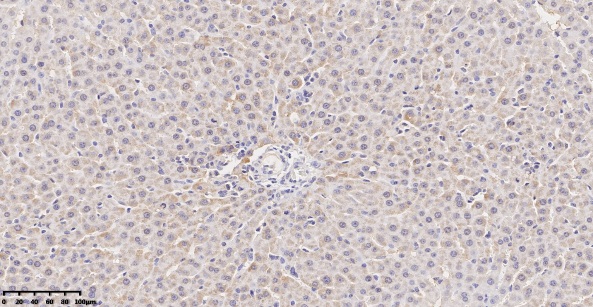

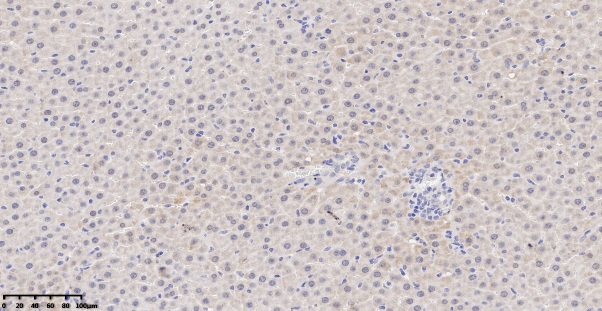
**

**MTOR:**

**Control: ANIT:**

**
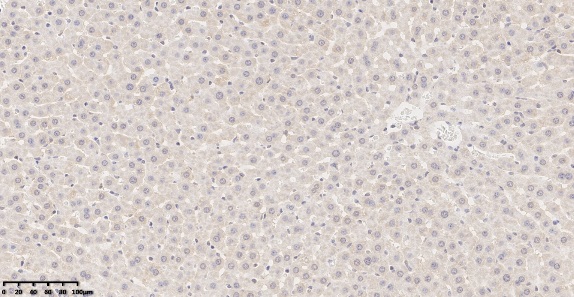

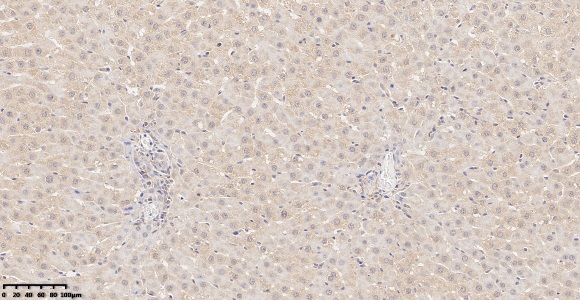
**

**PFL: PFH:**

**
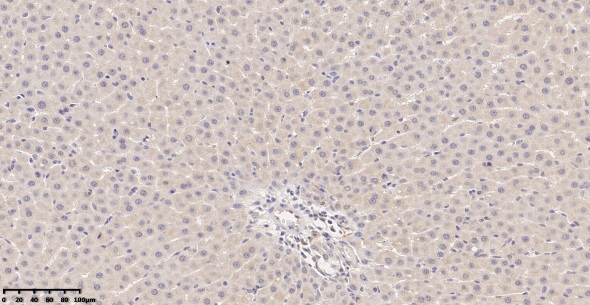

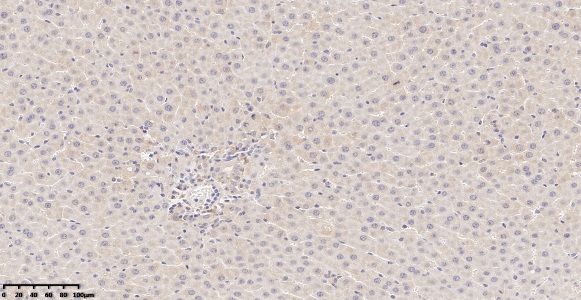
**
